# Compilation of Consolidation Properties Data of Champlain Sea Clay from Ottawa Region

**DOI:** 10.1007/s10706-024-02852-y

**Published:** 2024-07-10

**Authors:** N’eem Tavakkoli, Won Taek Oh, Sai K. Vanapalli

**Affiliations:** 1 Department of Civil Engineering, University of Ottawa, Ottawa, ON Canada; 2 Ministry of Transportation Ontario, Toronto, Canada; 3 Department of Civil Engineering, University of New Brunswick, Fredericton, NB Canada

**Keywords:** Champlain Sea clay, Sensitive clays, Consolidation, Soil database

## Abstract

Estimation of consolidation settlements in fine-grained soils due to various civil infrastructure loads is traditionally based on results derived from consolidation tests performed on undisturbed soil samples, combined with the data of other soil properties. In many geotechnical engineering applications, consolidation settlements are also estimated using empirical consolidation parameters derived from basic soil properties. This approach relies on correlations from the literature to bypass the time-consuming and expensive sampling techniques, laboratory testing, and other associated expenses. However, these correlations may not provide reasonable consolidation settlement estimations as these correlations are typically developed without considering the influence of stress history, geology, salinity of pore water, gradation, soil fabric, and chemical properties of the soils. This is especially true for Champlain Sea clay deposits from Eastern Ontario region of Canada that are typically with heterogeneous site conditions and exhibit spatial variability of soil properties. In this paper, data from the published literature and industrial and government reports on sensitive Champlain Sea clays were gathered for the Ottawa region. The data collection and clean-up methodology towards enhancing the reliability of the gathered data is comprehensively discussed. The summarized data from this study can be used with a greater degree of confidence towards developing reliable correlations in the estimation of consolidation settlements in geotechnical engineering practice.

## Introduction

The consolidation properties and corresponding parameters assessment of soft and sensitive clayey soils such as Champlain Sea clay of Eastern Canada poses significant challenges in geotechnical engineering practice. This is mainly attributed to the inherent heterogeneity and spatial variability in Champlain Sea clay deposits, and the challenges associated with expensive sampling and laboratory testing (Elkateb et al. 2003; Saye et al. 2021; Shi and Wang 2022). This is especially true for low or medium budget projects with limited resources for sampling and laboratory testing.

The terms “Champlain Sea clay”, “Leda clay”, or “Sensitive clays of Eastern Canada” are often used interchangeably in the literature. The soft and sensitive clay deposits in Eastern Canada initiate eastward from the rock terrains of the Canadian Shield, situated west of Ottawa. These deposits extend beyond Ottawa towards the eastern regions of Ontario and encompass a substantial expanse within Quebec (Chapman and Putnam 1984). Various case studies of buildings and other infrastructure damages due to excessive consolidation settlements that are constructed on Champlain Sea clay (hereafter referred to as CSC) deposits are annually reported in the Ottawa region. Frequent claims related to CSC problems triggered a comprehensive study by the City of Ottawa, to investigate these challenging soils. The report that was published in 2003 suggested from the investigations performed over a period of two years, that a notable section of the City of Ottawa is located on CSC deposits, which exhibit vulnerability to settlement when utilized as foundation materials. Four main contributors to the building damages that have been identified in this report, include: (i) climate change, (ii) urbanization in the region, (iii) vegetation, and (iv) unforeseen and uneven foundation loads (Lathrop and Leclair 2003). The first three factors contribute to a decrease in groundwater levels, subsequently leading to the occurrence of excessive consolidation settlements. On the other hand, the last factor is rooted in the overestimation of soil capacity at the design stage. In addition, soil shrinkage-induced damage to the buildings is also a significant concern in certain areas of Ottawa. These observations were supported in the last six decades as numerous residential structures situated on CSC deposits exhibit extensive cracks (e.g., Bozozuk 1962). However, in many cases, the influence of groundwater level decline in association with these environmental factors or other relevant activities are not accounted for in the foundation design (CBC 2012; City of Ottawa 2018). Bozozuk (1975) concluded that the groundwater level decline associated with urbanization is the major contributor to excessive consolidation settlement (e.g., Victoria Memorial Museum in Ottawa).

In July 2020, a local newspaper reported that the City of Ottawa had to buy out a total of 20 row houses due to excessive consolidation settlements caused by improper installation of municipal infrastructure. Such a decision had to be made to compensate for the losses of approximately $10 M CAD that was paid to various homeowners, over a period of a few years (Willing 2020). Two engineering consultants investigated this case and concluded that improper installation of a sewer trunk lowered the groundwater level, which contributed to the excessive consolidation settlements and subsequent damages to the houses (Genivar 2011; Stantec Consulting Ltd. 2014).

A two-story building located at 2001 Thurston Drive in Ottawa suffered severe cracks associated with excessive deformations. The forensic geotechnical investigation highlighted that the existing building was built on approximately 10 m of CSC deposit that contributed to the cracks in the building. Construction of a new structure close to this building which includes an underground parking garage had caused local groundwater level decline, which led to significant consolidation settlements (Stantec Consulting Ltd. 2012).

Bozozuk (1962) demonstrated that a decline in local groundwater levels, attributed to the presence of tall trees, can induce considerable non-uniform settlements. Such settlements, subsequently, have the potential to instigate damage and cracking in residential structures. These conclusions were derived from research studies focused on a ten by three city blocks area, involving a two-year groundwater level monitoring at selected locations. The monitoring outcomes indicated that the tall trees along the street perimeter resulted in a variation of 0.9 to 1.5 m in groundwater levels between the front and rear portion of the buildings.

Several new subdivisions were constructed with the rapid growth of the city of Ottawa. Many of these subdivisions are built by converting the farmlands and forests into residential areas. Excessive consolidation settlements were observed in several of these subdivisions as many buildings were built in CSC deposits that contributed to the lowering of the natural groundwater level. For this reason, it is essential to consider the influence of variations in groundwater levels in their designs and assessments to minimize the risk of instability and excessive consolidation settlement of structures. However, even if the groundwater level variations are considered during design stage, the excessive consolidation settlements may still occur due to the possible errors in the empirical estimations of consolidation parameters, that include the compression index, recompression index, and preconsolidation pressure. It is common geotechnical engineering practice, especially for smaller projects with low budget, opting to exclude consolidation testing results. In other words, consulting firms routinely generate their reports using limited information, alleviating comprehensive consolidation test data that are required for reliable estimation of settlements in sensitive clays. Due to these reasons, excessive and uneven consolidation settlements are likely to arise and damage buildings, in several scenarios.

In the present study, an effort was made to collect consolidation parameter data from the Ottawa region. This involved utilizing over 500 foundation engineering reports, including those prepared for the Ontario Ministry of Transportation. The collected data was cleaned up and analyzed statistically based on skewness and kurtosis. In addition, spatial distribution of consolidation parameters data was also investigated. Comprehensive details of clean-up methodology and statistical analyses of the data are summarized in later sections of the paper.

## Champlain Sea Clay (CSC)

The CSC deposits are postglacial marine sediments that cover most of the St. Lawrence lowlands in Eastern Canada (Gillott 1970). Various processes such as flow turbidity, basin salinity, geochemical factors, and temperature have greatly influenced the stratigraphy and dispersion of Champlain Sea clay (i.e., CSC) during sedimentation. These clay deposits, despite being generic referred by one name, demonstrate noticeable differences in composition and mechanical behavior across the region mainly due to the varying salt concentrations (Laventure and Warkentin 1965; Quigley 1980). The main source of these deposits are igneous and metamorphic rocks of the Canadian Shield. Mica and chlorite are the predominant minerals of Champlain Sea clay and amphibole, quartz, and feldspar are found in smaller proportions (Laventure and Warkentin 1965).

The age of CSC deposits in different basins may vary between 5,000 to 12,000 BP (Demers and Leroueil 2002). The ice sheet retreat occurred from south to north. As the glacier receded, the Champlain Sea formed in its place. Hence, southern regions host aged CSC deposits, whereas their younger counterparts are predominantly located in the north. Champlain Sea clays were generally deposited within brackish water resulting from the mix of freshwater ice melt and intruding water from the rising Atlantic Ocean (Haché et al. 2015). Varved clays are formed in the freshwater lakes whereas in the coastal areas saline water entered the basins and flocculated the sediments.

CSC deposits exhibit complexity in nature and are prone to strength loss and restructuring (Laventure and Warkentin 1965; Gillott 1970; Agaiby and Mayne 2018; Mayne et al. 2019), primarily due to their inherent high sensitivity (i.e., quick clay conditions). The salinity of porewater, gradation, soil fabric and coarseness, chemical properties, and electrokinetic potential are all important factors that contribute to the sensitivity of CSC (Liu et al. 2020). The liquid limit (*LI*) and plasticity index (*PI*) decrease with increasing salinity (Johny et al. 2024). For example, according to the experiments conducted by Liu et al. (2020), a decline in salinity from 9.50 to 0.35 g/l resulted in a decrease in the plasticity index (*PI*) by 14 to 31% and in the liquidity index (*LI*) ranging from 4 to 26%. Leaching weakens bonding between particles (Saadatkhah et al. 2023; Takahashi and Kanayama 2023), elevating sensitivity and compressibility by approximately 20%, and simultaneously contributing to a reduction in the preconsolidation pressure by 30%. The salinity of CSC porewater is generally less than 2 g/l (Gillott 1970); however, it can vary from 4 to 21 g/l, typically increasing with depth (Liu et al. 2020). While salinity is not routinely assessed in conventional geotechnical engineering practice, it provides valuable information to understand the variations of consolidation characteristics within CSC deposits across the region.

**Fig. 1:**
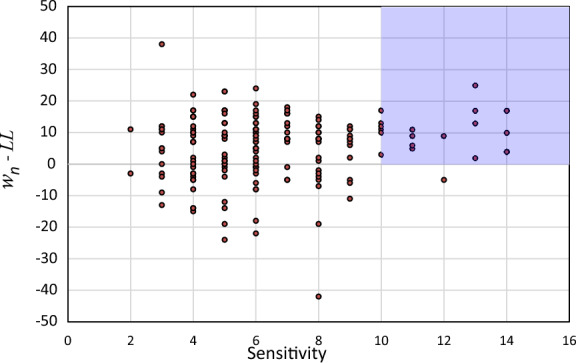
Plot of the sensitivity vs. the difference between natural water content and liquid limit using the database compiled for this study

One characteristic of sensitive clays is their high natural water content (*w*_*n*_), that can exceed the soil’s liquid limit (*LL*). According to the database compiled for this study, 194 out of 279 samples had *w*_*n*_ that exceeded *LL* by 11%, with an average sensitivity of 6.7. On the other hand, 85 samples had *w*_*n*_ less than *LL* by 8%, with an average sensitivity of 5.8. From Fig. 1, it can be derived that the samples with high sensitivity (≥ 10) show *w*_*n*_ greater than *LL*.

**Fig. 2:**
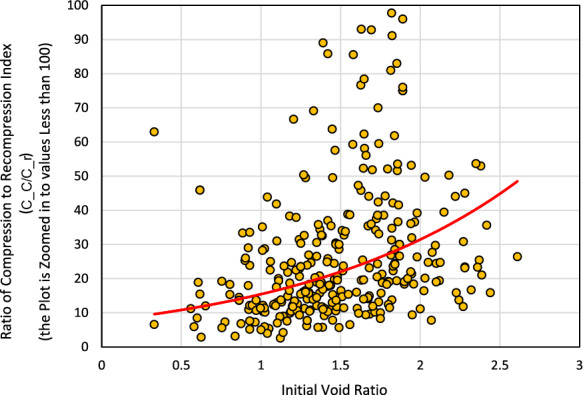
Ratio of compression index to recompression index vs. initial void ratio using the database compiled for this study

The undisturbed structure of very sensitive or quick clays is typically open and flocculated (Jarrett 1972) with high void ratio. This flocculated structure with high initial void ratio triggers sensitive behaviors, which leads to noticeable restructuring and high compression when stressed beyond their preconsolidation pressure. Due to this reason, the ratio of compression index (*C*_*c*_) to the recompression index (*C*_*r*_) increases with an increase in the initial void ratio, as shown in Fig. 2 based on the database compiled for this study.

## Problem Statement

The common techniques used in the estimation of consolidation settlements in practice are simple and straightforward (Craig 2004; Budhu 2010; CFEM 2023). Several software packages are also available to assist in the estimation of the consolidation settlements for complex scenarios. The consolidation settlement is commonly estimated using the basic soil parameters (i.e., soil density, *γ*, void ratio, *e*, water content, *w*, specific gravity, *G*_*s*_) and those determined from oedometer tests on undisturbed samples (i.e., compression index, *C*_*c*_, rebound or swelling index, *C*_*r*_ (also abbreviated as *C*_*s*_), and the pre-consolidation pressure, $$\sigma_{P}^{\prime}$$). The undisturbed samples required for oedometer tests are typically collected using thin-walled tubes from the field. Special piston samplers are necessary to collect highly sensitive clay samples. However, despite comprehensive theoretical background and recommended procedures, practitioners commonly use correlations from the literature to estimate consolidation parameters based on simple laboratory tests. Reliable settlement estimations are not likely due to the lack of comprehensive consolidation test data, particularly in sensitive clay environments. The practice of using empirical procedures stems from the high costs associated with extensive field investigations and laboratory testing, which are significantly higher than the costs of engineering analysis and reporting. As per estimations undertaken in 2023, the cost for each consolidation test in Ontario is in the range from $1,900 to $2,300 CAD, which is about 30% of the typical geotechnical engineering fees for low budget projects such as the residential buildings (Tavakkoli 2023).

Numerous correlations for estimation of consolidation settlement have been established through decades of research for different soils and have been widely used in geotechnical engineering practice (Bjerrum 1972; Tavenas et al. 1974; Bjerrum and Aitchison 1975; Tavenas and Leroueil 1979, 1987; Leroueil et al. 1983; Chang 1991; Mayne 1992; Robertson et al. 1992; Chen 1994; Chen and Mayne 1996; Demers and Leroueil 2002; Elkateb et al. 2003; McQueen et al. 2016; Agaiby and Mayne 2018; Mayne et al. 2019). However, limited data is available from published literature to establish reliable correlations for CSC, which is more challenging due to the drastic changes in the properties of CSC across the vast clay plains. For this reason, it is necessary to refine the existing correlations based on specific zoning, considering the various geographical micro-zones. Recently, there have been improvements in terms of access to interactive surficial geology maps and online borehole libraries with the capabilities of visually locating the closest historical records to the project site (ENDM 2024). Nevertheless, the data information that is currently available primarily offers a generic identification of stratigraphy; however, it lacks the synthesized information required for the development of a dependable geotechnical model. In other words, historical data is summarized as discrete pieces of information. The excessive use of generic approximations without a thorough understanding, based on discrete pieces of information and ignoring site-specific geotechnical conditions, can lead to overdesign or serviceability failure, especially in low-budget projects. Moreover, as the scale, significance, and impact of a project increases, typically, a more comprehensive and detailed site-specific testing is required.

For this reason, the present study is structured to focus on elucidating a methodology for rational summarization of consolidation parameters data collection within the Ottawa area. It is expected that this study will be valuable for use in geotechnical engineering practice applications for CSC deposits in Eastern Ontario, Canada, particularly within the Ottawa region.

## Data Collection and Database Structure

In this study, basic soil properties and consolidation parameters from numerous geotechnical reports generated by the industry in recent years have been compiled. Most of the compiled data are related to the design of foundations in the soft and sensitive clays in Eastern Ontario, and mostly within the limits of the City of Ottawa. While gathering data, it became clear that a significant number of the reports utilized guidelines from building codes for approximate estimation of soil bearing capacity based on soil classification information for the design of foundations instead of conducting direct and quantitative field/laboratory testing. While this method is accepted in geotechnical engineering practice in several scenarios, relying solely on the guidelines might diminish design precision due to substantial variability in soil parameters across the region. Such design guidelines are approximate estimations and do not consider many factors such as the clay thickness, groundwater table, sensitivity, and consolidation parameters, to list a few.

As previously noted, the primary data for this study was derived from industry-prepared reports. In Canada, the Ministry of Transportation Ontario (MTO) releases reports under the ‘Foundation Library’, renowned for adhering to the highest industry standards in their preparation (MTO 2020). Also, reports prepared for larger municipalities are considered reliable sources due to their perceived high level of accuracy and detailed information (City of Ottawa 2024). In addition, several academic publications with datasets on CSC were also used (e.g., Konrad 1987; Konrad and Law 1987a, b; Lafleur et al. 1988; Chen and Mayne 1994). Details related to the quantity of collected reports used in the study are summarized in Table 1.

**Table 1:** Quantity of the foundation engineering reports collected from Ottawa region

MTO foundation reports on clay (cell A)	Number of reports with consolidation information	107
(cell B) - Please merge cell A & B	Number of reports with no consolidation information	238
Other foundation reports on clay	Number of reports with consolidation information	20
	Number of reports with no consolidation information	139
Research reports with consolidation information		5
Total		509

### Zoning for Data Collection

In Ontario, CSC deposits are mostly found within the Ottawa Valley, and large basins of this clay exist within the Ottawa region. The extent of the clay plains is mostly observed from Pembroke to Hawkesbury. The clay plain is intermittently interrupted by rock or sand ridges. Within the valley, some of the uplifted faulted bedrock blocks emerge above the clay bed (Chapman and Putnam 1984) and the ground slope is relatively gentle south of the Ottawa River (compared to the Quebec side, north of the river). The sediments are noted to be deep silty clays. The depth of clay deposits drastically varies across the clay plain depending on the geological setting.

**Fig. 3:**
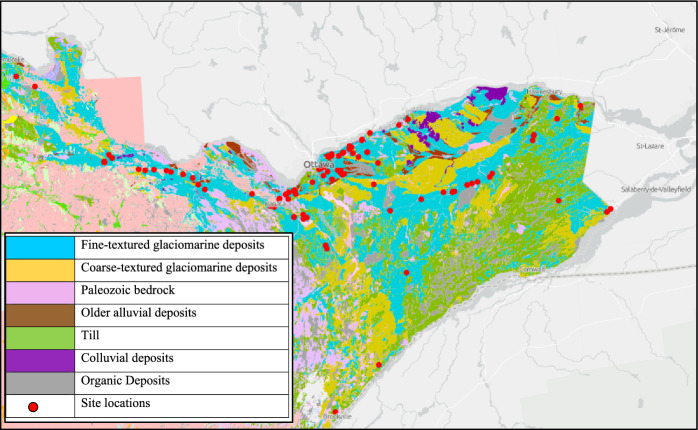
Surficial geology polygons of Eastern Ontario with site locations (red circles)

Among various surficial geology sources available, for this research, the Quaternary geology prepared by the Ontario Geological Survey and published by the Ministry of Northern Development, Mines, Natural Resources and Forestry was used as the base mapping to define the areas covered by clay plains (ENDM 2024). The geology polygons superimposed on Google Earth are shown in Fig. 3. As can be seen, the Ottawa Valley region is characterized by the predominant presence of the CSC (in dark blue) in its surficial geology, extending to the south and east. Notably, the CSC does not extend substantially to the west of Ottawa. The clay plain is frequently interrupted by finer glacial till deposits (in green polygons), and coarser sand and gravel (in yellow). Rock outcroppings are depicted in pink. The dark brown and gray depict loose alluvial deposits and organic sediments at the surface, respectively. As most of the data is sourced from the MTO Foundation Library, reports primarily focus on the locations along the major provincial highways and roads, such as Hwy 417, Hwy 174, and Hwy 17 East.

**Fig. 4:**
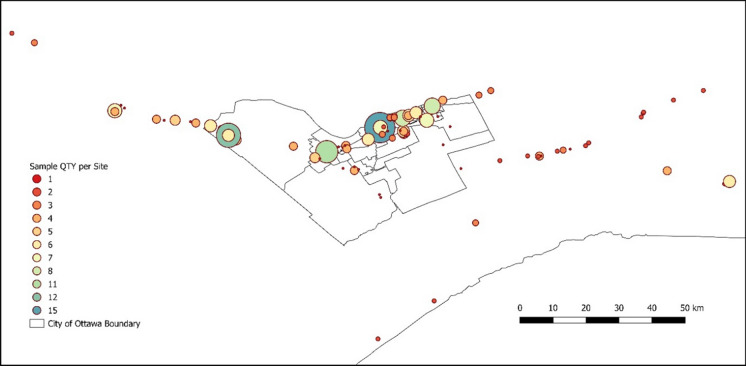
Site Locations. The number of consolidation tests per site are highlighted with the size of the circles (*Note* the larger the circle, greater is the number of consolidation tests)

**Fig. 5:**
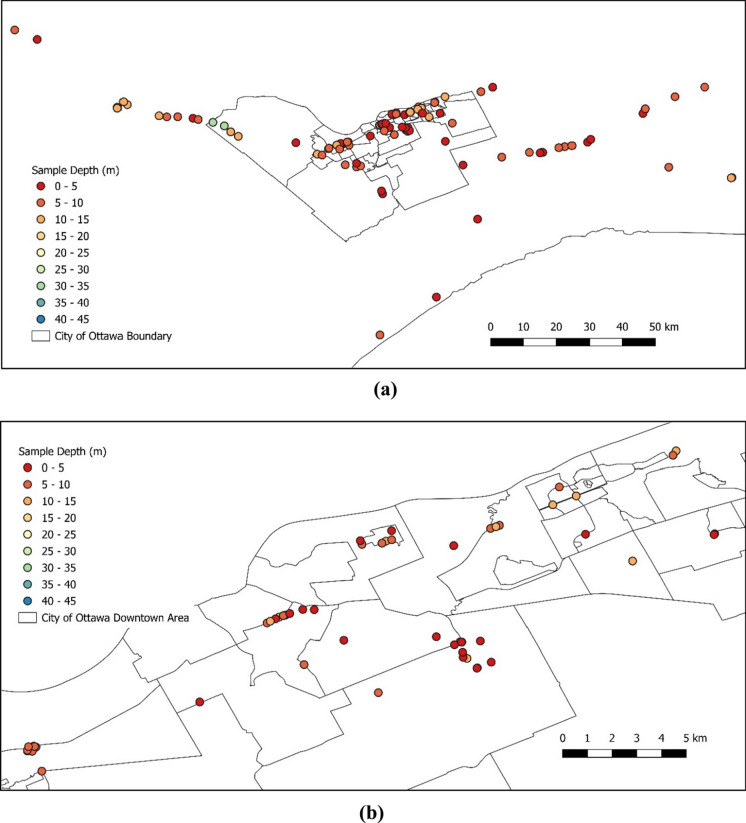
Distribution of sample depths **a** zoomed out image of sample depths across Ottawa area, **b** a focused image of sample depths and site locations within the Ottawa lower town area

Most of the reports featured just one consolidation test; however, some reports incorporated multiple tests conducted for a singular project. The relative quantity of data points collected for each site is shown in Fig. 4, which is highlighted by the size of the circles for each site for visual comprehension. As can be seen, the concentration of data within the City of Ottawa limits suggests that the synthesized data can be regarded as representative of a certain locality. Figure 5 shows the distribution of sampling depths across the Ottawa region, which are relatively consistent within the upper 10 m from the surface with few exceptions collected from deeper than 15 m.

### Data Structure and Extraction from Existing Reports

A total number of 509 foundation engineering reports have been collected for the Ottawa region, including MTO reports, research reports, and ‘other’ reports, as shown in Table 1. ‘Other’ reports include those prepared for both municipalities and private sectors. Among those reports, 132 reports included information on consolidation testing. When raw data was unavailable, measurements were gathered using the scaled measuring tools from images in the reports. All units were converted to a consistent metric system (Table 2).

**Table 2:** Database structure

Data category	Data entry field	Unit
General laboratory Index testing	Natural water content (${w}_{n}$)	%
	Liquid limit ($LL$)	%
	Plasticity index ($PI$)	%
	Specific gravity (${G}_{s}$)	n/a
	Total unit weight (${\gamma }_{t}$)	kN/m^3^
	Silt percentage ($M$)	%
	Clay percentage ($C$)	%
Field testing	Depth ($d$)	m
	Undrained shear strength (${S}_{u}$)	kPa
	Sensitivity (${S}_{t}$)	n/a
Consolidation laboratory testing	Effective in-situ stress (${\sigma }_{o}^{\prime}$)	kPa
	Preconsolidation pressure (${\sigma }_{P}^{\prime}$)	kPa
	Overconsolidation ratio ($OCR$)	n/a
	Compression index (${C}_{c}$)	n/a
	Recompression index (${C}_{r}$)	n/a
	Coefficient of consolidation (${c}_{v}$)	mm^2^/s
	Initial void ratio (${e}_{o}$)	n/a

Consolidation graphs (i.e., *e* – log*σ’* curve), in most reports, were presented with a few missing information such as *C*_*r*_ and *c*_*v*_. Although *C*_*r*_ could be estimated based on consolidation graphs, *c*_*v*_ could be retrieved only from the values that are documented in the reports. Most reports required cross-referencing of test results with borehole logs or confirming the calculated values based on laboratory test results as much as possible with the available information. The following conventions were applied throughout the data entry process.

- Natural water content and plasticity parameters are rounded to the full number to provide consistency across the database.
- In cases where the preconsolidation pressure was not available, it was determined using the Casagrande graphical method (Casagrande 1936).
- A large proportion of reports contained unit weight values. Where this information was missing, an average value of 16.8 kN/m^3^ was used to calculate the in-situ stress. This is the average value from the documented entries of the database for unit weight. It is also consistent with the value measured from the Ottawa city Gloucester Test Site from tube samples (Mayne et al. 2019). Also, when feasible, the unit weight was back-calculated by utilizing initial void ratio and specific gravity with mass-volume relationships. Many reports opted for a commonly accepted range of specific gravity values (i.e., between 2.72 to 2.76) rather than determining the value from laboratory tests.
- The utilized *c*_*v*_ values align with the stress level equivalent to the estimated preconsolidation pressure of the corresponding test.
- The in-situ stress was recalculated using the data from the borehole logs if the in-situ stress reported in the consolidation graphs conflicted with the information summarized in borehole logs.
- For soils characterized by high silt content and a lower *OCR*, approximately equal to 1, leading to a narrow range of overconsolidation, *C*_*r*_ was derived from the unloading curve.
- In certain instances, the calculated in-situ stress was marginally and insignificantly lower than the reported preconsolidation pressure. In such situations, the *OCR* was documented as equal to 1.

**Fig. 6:**
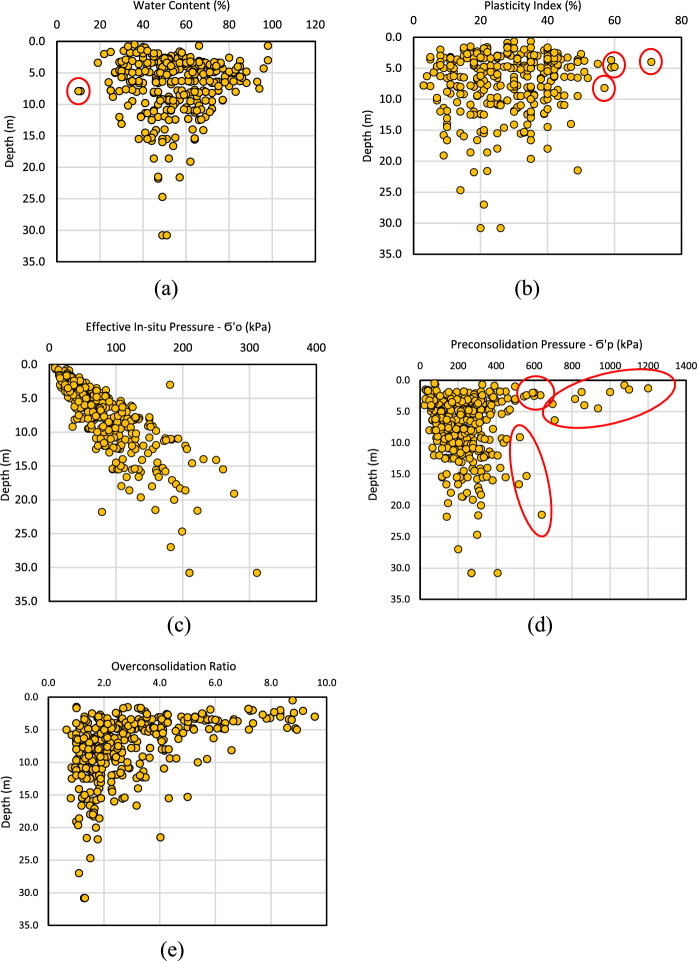
Distribution of **a** natural water content, **b** plasticity index, **c** effective stress, **d** preconsolidation pressure, and **e** overconsolidation ratio with sampling depth across the Ottawa area (The data in the red circle indicate outliers)

Distributions of natural water content, plasticity index, effective stress, preconsolidation pressure, and overconsolidation ratio with sample depths across the Ottawa area are shown in Fig. 6a–e. The data marked with a red circle indicates outliers identified by the statistical analyses in the present study. The coefficient of consolidation, *c*_*v*_ was included in the data collection only when it was provided in the foundation reports.

Efforts were made to gather information related to the thickness of clay layer. However, it became apparent during data collection that the number of boreholes providing such information was relatively small compared to the total borehole dataset collected. As a result, the data summarized by authors may not accurately represent the geophysical conditions of the study area. This limitation primarily arises from the standard practice in geotechnical engineering, where borehole drilling is typically halted once the presumed influence zone of stresses associated with the footing loads is reached. Exceptions however were observed for structures like bridges and taller buildings, which require a more stable stratum such as till or bedrock.

### Statistics of the Database

In the preliminary phase, statistical summaries were generated for the raw data set. After the initial statistical analysis, it became evident that the implementation of a data clean-up program was imperative. The cleanup methodology includes eliminating outliers from each parameter dataset. The outliers were identified based on their deviation from the median values. The three data quartiles were identified for each dataset. Knowing the second quartile, *Q*_*2*_, represents the middle value, the range between the first and third quartile is referred to as Interquartile Range (*IQR*). In this context, data beyond 1.5 times the *IQR* was defined as an outlier. Data clean-up and the outlier concept were not applied to some of the datasets such as soil sampling depth, and the in-situ effective stress. Skewness and kurtosis were however considered for all the parameters. Skewness is a measure of distribution asymmetry of the normal distribution curve (Eq. (1)) and kurtosis of a dataset shows its peakedness or flatness compared to normal distribution (Eq. (2)).

1 $$Skewness=\frac{n{\sum }_{i=1}^{n}{\left({x}_{i}-\overline{x }\right)}^{3}}{\left(n-1\right)\left(n-2\right){\delta }^{3}}$$

2 $$Kurtosis=\left\{\frac{n\left(n+1\right)}{\left(n-1\right)\left(n-2\right)\left(n-3\right)}\sum {\left(\frac{{x}_{i}-\overline{x}}{\delta }\right)}^{4}\right\}-\frac{3{\left(n-1\right)}^{2}}{\left(n-2\right)\left(n-3\right)}$$

where *δ* = standard deviation, *n* = number of variables, $\overline{x }$ = mean value, and ${x}_{i}$ = variable from *i* to *n*.

For data cleanup, skewness range of ± 2 and kurtosis range of ± 7 were considered as the acceptable range of deviation from normal distribution (Byrne 2010; Hair et al. 2010). Acknowledging the inherent variability in accuracy and precision of industry-generated reports, it is important to note that this diversity can have adverse implications for the establishment of correlations during the processes of data collection, analysis, and reporting. Hence, reports containing ambiguous or conflicting information were excluded during the data entry phase.

### Elimination of Outliers

An outlier is an observation which drastically deviates from other observations in a way that arises suspicions (Hawkins 1980). It is crucial to identify and subsequently remove the outliers since experimental data might be prone to significant human and environmental errors (Wang and Vanapalli 2024). Abnormal data can be associated with erroneous measurements, misreporting, sampling errors or computational errors. If incorrect observations cannot be methodically corrected, then they must be eliminated (Domański 2020). A judgment call may be made for removing or retaining the outliers. One major obstacle in assessing the legitimacy of the outliers for potential retention is the uncontrolled data sampling and collection procedure. For example, the data used in the present study was generated over a long period of time by numerous operators, various tools and measurement accuracy level, and different approaches towards quality control, which are normally observed slightly different from one consulting firm to another. Hence, in the present study, the data was analyzed using skewness and kurtosis, which are unbiased statistical methodology to detect outliers.

## Analyses of Dataset

**Fig. 7:**
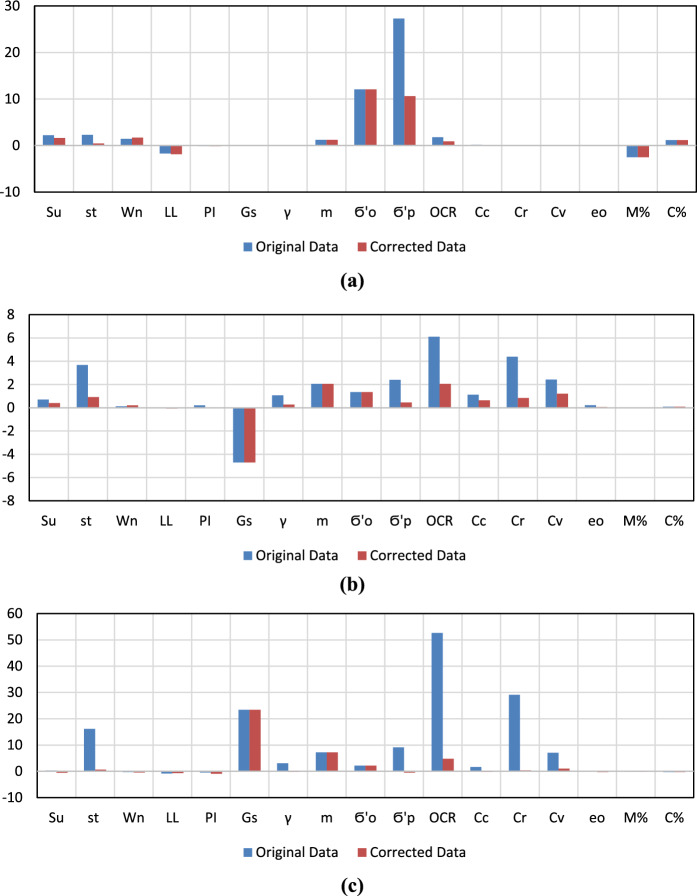
Improvement of statistical indices after data cleanup; **a** difference between average and median, **b** data skewness, and **c** data kurtosis

Table 3 summarizes raw data that includes the quantity of each parameter and the statistical distribution. Comparisons between the original (upper row) and cleaned-up (lower row) database statistics are shown in Table 4. Difference between the average and the median, data skewness, and data kurtosis for the soil properties summarized in Table 4 are visually demonstrated in Fig. 7a–c, respectively.

**Table 3:** Statistics of raw data

Parameter	Qty	Min	Max	Ave	Med	Skew	Kurt
Depth (m)	417	0.5	42	7.3	6.1	2.057	7.234
Natural water content, ${w}_{n} \left( \% \right)$	335	10	98	54	53	0.121	− 0.297
Liquid limit, ${LL} (\text{\%})$	279	18	97	50	52	− 0.005	− 0.820
Plasticity index, $PI (\text{\%})$	332	3	71	27	28	0.201	− 0.446
Specific gravity, ${G}_{s}$	28	2.18	2.8	2.74	2.76	− 4.694	23.415
Total unit weight, ${\gamma }_{t} \left(\text{kN}/{\text{m}}^{3}\right)$	275	13.5	23.3	16.8	16.7	1.071	3.069
Silt percentage, $M (\text{\%}$)	80	13	88	43	46	0.028	− 0.089
Clay percentage, $C(\text{\%})$	80	4	99	49	48	0.098	− 0.298
Undrained shear strength, ${S}_{u} (\text{kPa})$	285	12	148	54	52	0.701	0.345
Sensitivity, ${S}_{t}$	222	2	55	8	6	3.678	16.178
Effective overburden pressure, ${\sigma }_{o}^{\prime} (\text{kPa})$	427	8.6	311	80	68	1.352	2.211
Preconsolidation pressure, ${\sigma }_{P}^{\prime} (\text{kPa})$	427	24	1200	236	209	2.397	9.103
Overconsolidation ratio, $OCR$	425	0.7	76.8	4.3	2.5	6.110	52.667
Compression index, ${C}_{c}$	364	0.048	4.101	1.076	0.920	1.115	1.645
Recompression index, ${C}_{r}$	364	0.02	0.510	0.0557	0.043	4.383	29.097
Coefficient of consolidation, ${c}_{v} \left({\text{mm}}^{2}/\text{s}\right)$	58	0.005	1.828	0.294	0.194	2.425	7.066
Initial void ratio, ${e}_{o}$	369	0.33	3.036	1.5305	1.48	0.225	0.069

**Table 4:** Comparison between the original (upper row) and clean-up (lower row) database statistics

	Depth (m)	$S_{u}$ $\left( {{\text{kPa}}} \right)$	$S_{t}$	*w*_*n*_ (%)	*LL* (%)	*PI* (%)	*G*_*s*_	$\gamma_{t}$ $\left( {{\text{kN/m}}^{{3}} } \right)$	$\sigma_{o}{\prime}$(kPa)	$\sigma_{P}{\prime}$(kPa)	*OCR*	*C*_*c*_	*C*_*r*_	*c*_*v* (mm2/s)_	*e*_*o*_	*M* (%)	*C* (%)
QTY	417	285	222	335	279	332	28	275	427	427	425	364	364	58	369	80	80
	417	279	202	333	278	327	28	269	427	400	400	354	340	54	366	80	80
Minimum	0.5	12	2	10	0	3	2.18	13.5	8.55	24	0.7	0.048	0.002	0.005	0.33	13	4
	0.5	12	2	19	18	3	2.18	13.5	8.55	40	0.7	0.048	0.002	0.005	0.33	13	4
Maximum	42	148	55	98	97	71	2.8	23.3	311	1200	76.8	4.101	0.510	1.828	3.04	88	99
	42	115	14	98	97	55	2.8	19.8	311	485	16.1	2.514	0.130	0.806	2.69	88	99
Average	7.3	54	8	54	50	27	2.74	16.8	80.1	236.3	4.3	1.076	0.056	0.294	1.53	43	49
	7.3	53	6	55	50	27	2.74	16.7	80.1	210.6	3.3	1.021	0.045	0.219	1.52	43	49
Median	6.1	52	6	53	52	27.5	2.764	16.7	68	209	2.5	0.920	0.043	0.194	1.48	46	48
	6.1	51	6	53	52	27	2.764	16.6	68	200	2.4	0.905	0.041	0.183	1.48	46	48
Skewness	2.1	0.7	3.7	0.1	0.0	0.2	− 4.7	1.1	1.4	2.4	6.1	1.1	4.4	2.4	0.2	0.0	0.1
	2.1	0.4	0.9	0.2	− 0.1	0.0	− 4.7	0.3	1.4	0.5	2.1	0.6	0.8	1.2	0.1	0.0	0.1
Kurtosis	7.2	0.3	16.2	− 0.3	− 0.8	− 0.4	23.4	3.1	2.2	9.1	52.7	1.6	29.1	7.1	0.1	− 0.1	− 0.3
	7.2	− 0.5	0.6	− 0.4	− 0.7	− 0.9	23.4	− 0.2	2.2	− 0.5	4.8	− 0.2	0.4	1.0	− 0.3	− 0.1	− 0.3
Ave.—Med	1.2	2.2	2.3	1.4	− 1.7	− 0.1	0.0	0.1	12.1	27.3	1.8	0.2	0.0	0.1	0.1	− 2.5	1.2
	1.2	1.6	0.5	1.7	− 1.9	− 0.1	0.0	0.1	12.1	10.6	0.9	0.1	0.0	0.0	0.0	− 2.5	1.2

The skewness and kurtosis of the raw data sets were found to be beyond the acceptable range for some parameters. High kurtosis values not only reflect the peaks of the distribution curve, but also result in flatter tails of the distribution which is the result of more frequent extremely high and very low values. Skewness is a measure of asymmetry in normal distribution. A distribution can exhibit right (positive), left (negative), or zero skewness. In a right-skewed distribution, the tail extends further to the right of its peak, while in a left-skewed distribution, the tail extends further to the left. The right skew is synonymous with positive skew, and left skew is synonymous with negative skew.

**Fig. 8:**
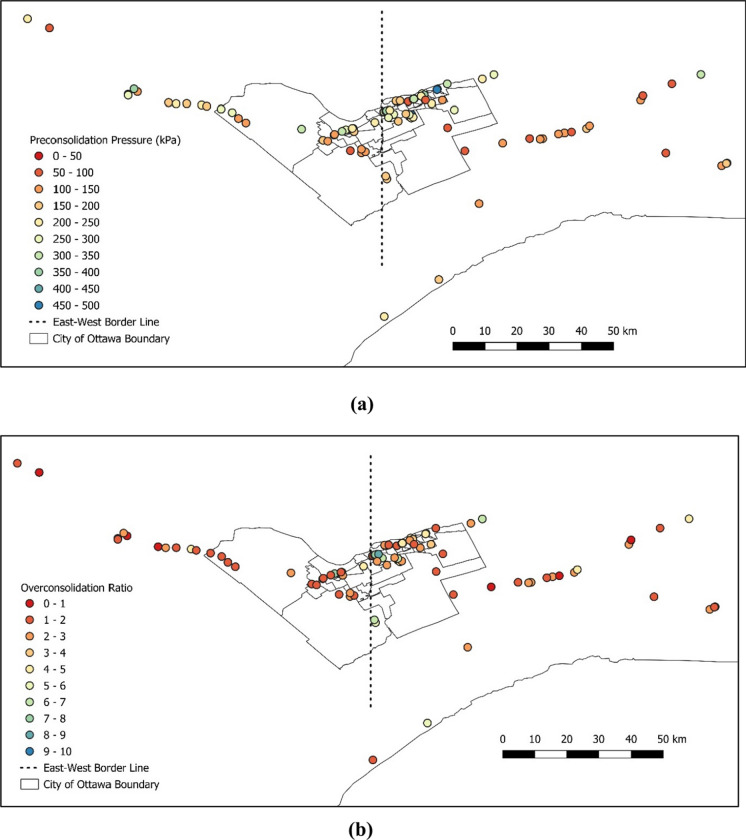

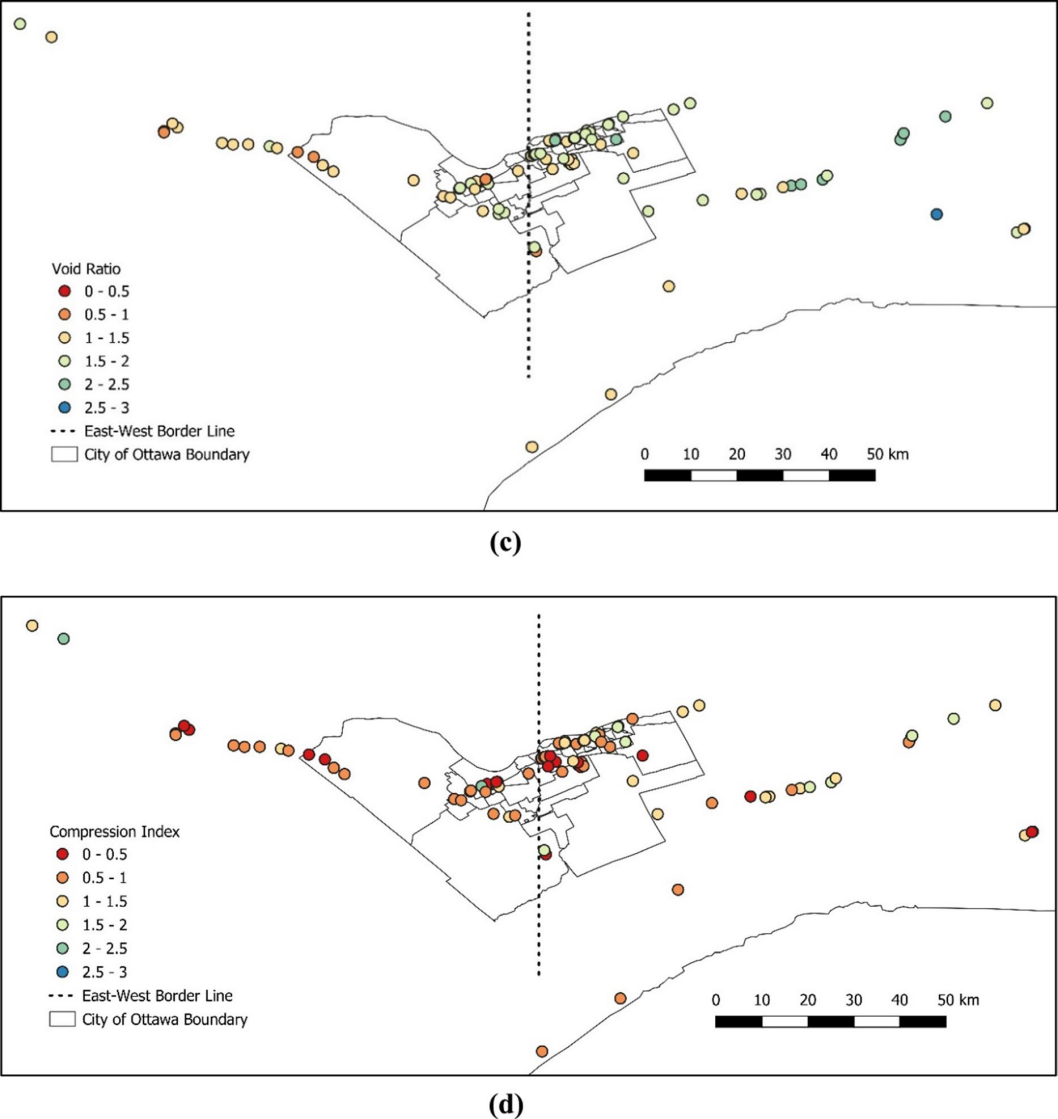

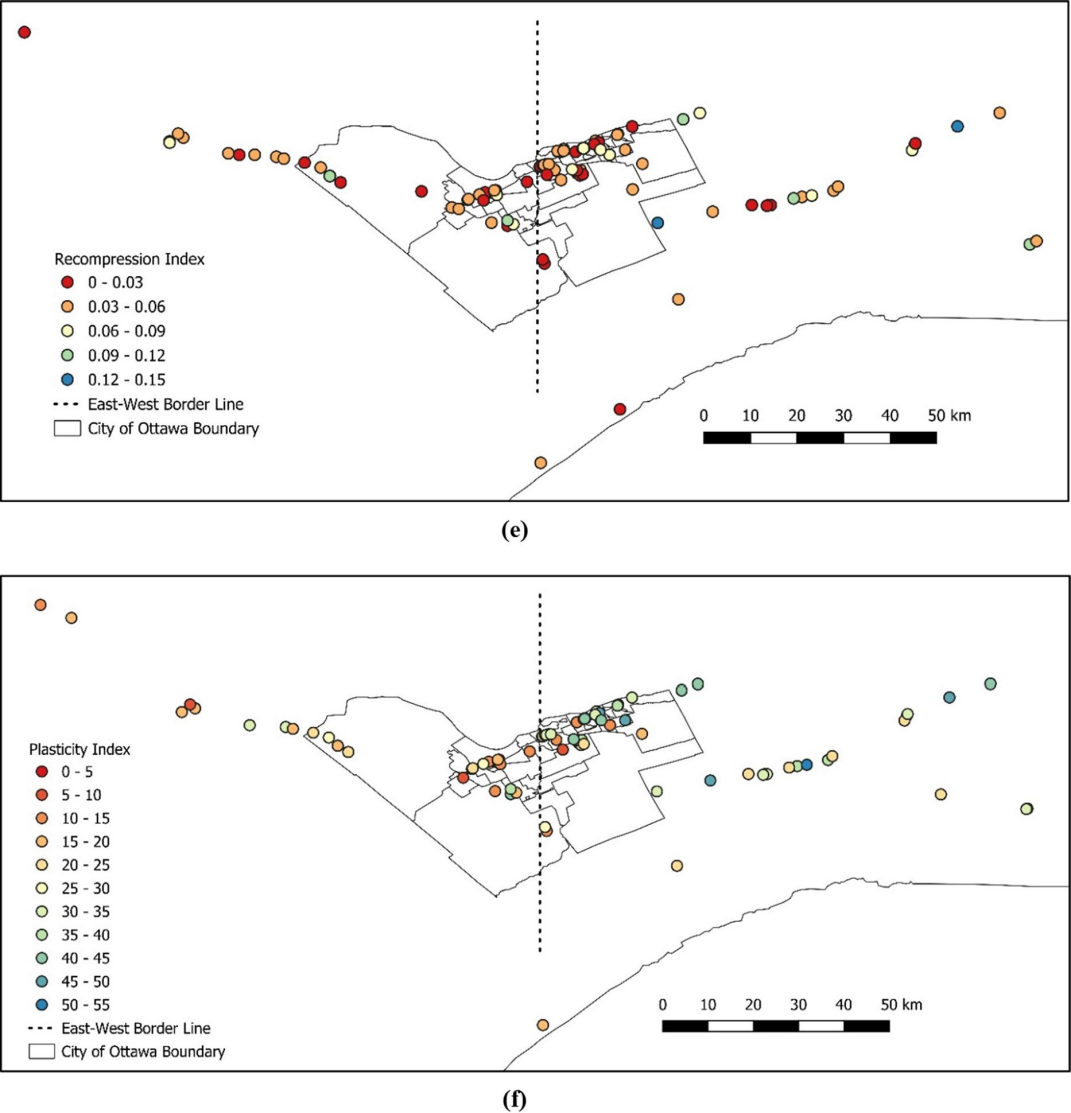

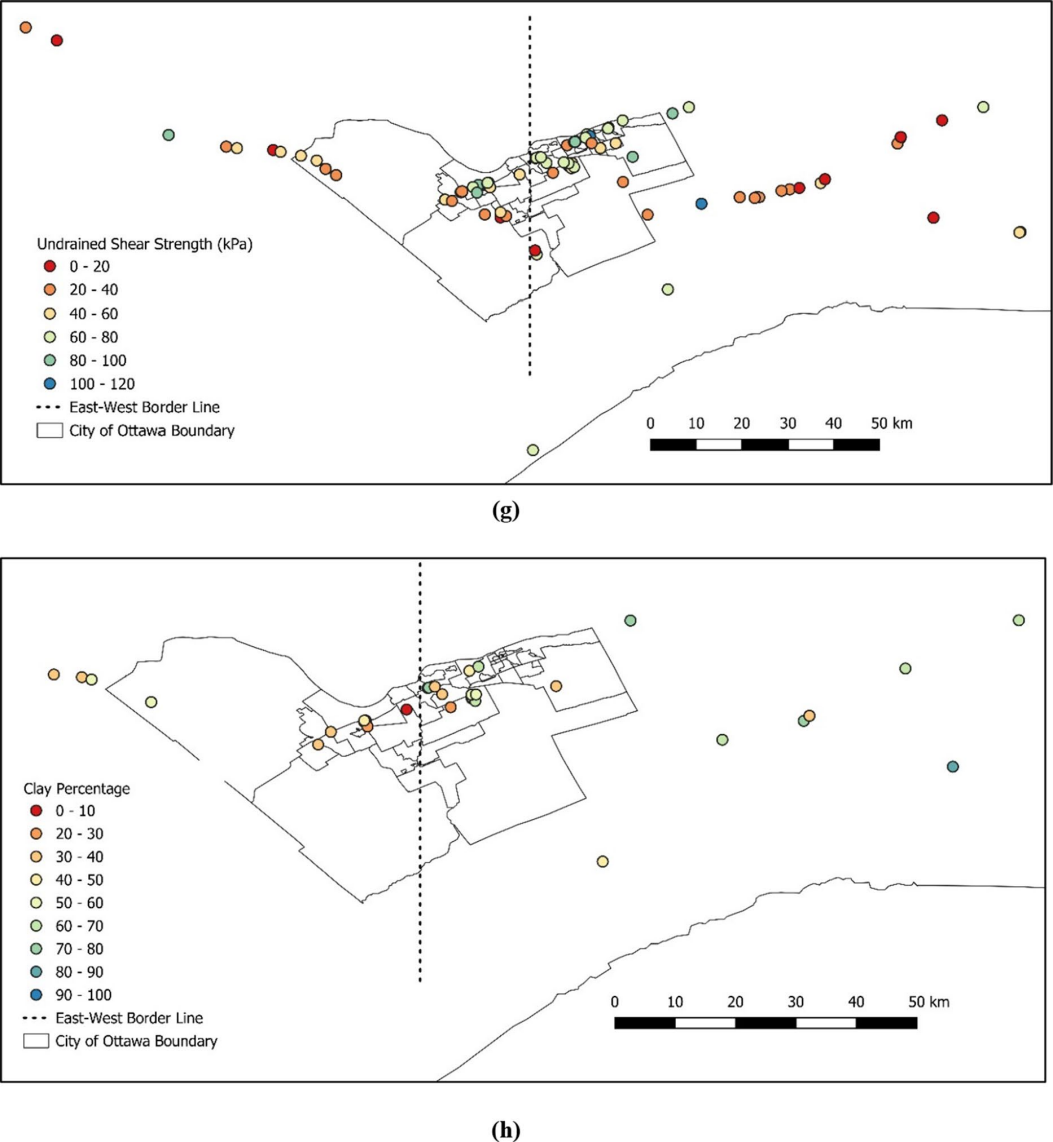
Characterization of certain parameters across the Ottawa area for **a** preconsolidation pressure, **b** overconsolidation ratio, **c** initial void ratio, **d** compression index, **e** recompression index, **f** plasticity index, **g** undrained shear strength, and **h** clay percentage

Once the data was cleaned up, the following data processing and spatial analysis were completed on several parameters as shown in Fig. 8a–h. This is for the purpose of visually evaluating the changes of various parameters across the area.

## Discussions

Several observations were made over the spatial plotting of various parameters, which are further examined here. The summarized discussion is presented in two parts; the first part discusses the statistical anomalies and variations observed during data collection, and the second part provides a discussion on the observations based on special data distribution.

### Statistical Variations of the Database

The anomalies observed in some of the statistical parameters can be attributed to the wide span of data generation. The data gathered in this study is generated over a long period of time (i.e., from 1950 to 2022) by different technical personnel or operators following different protocols, guidelines, and industry standards. It is noteworthy that some tests can be potentially more prone to operator error or bias than others. The source of bias also will not be of the same nature. A test such as the measurement of natural water content is less prone to operator bias in comparison to, for example, Atterberg limit tests that require more operator training. One obvious comparison can be made between the undrained shear strength and the sensitivity. Although a drastic difference is observed between the statistical markers for these two parameters, these two readings are typically made at the same time and the same depth by the same operators. The collected original data indicated a skewness of 0.7 and 3.7 and kurtosis of 0.3 and 16.2 for undrained shear strength and sensitivity, respectively. Based on the description of field operations reflected in the geotechnical reports, there is a consistency of practice for measuring the undrained shear strength, and the process is less prone to operators bias. One of the possible reasons for the drastic variations in recorded sensitivity (i.e., out of acceptable range of kurtosis) may be attributed to the operator bias. For example, the shear strength testing for remolded condition is performed differently with respect to the number of rotations to disturb the soil and the time for the soil to gain strength. According to Rosenqvist (1953), CSC is classified as a very sensitive material (Rosenqvist 1953) with an average sensitivity of 6, even after the data clean up. The sensitive clays can undergo sudden reduction in void ratio within a narrow change of effective stress (Scott 1989).

Laboratory testing for measurement of specific gravity is also a test which requires trained operators. Air bubbles from the test sample should be removed for obtaining reliable test results. However, in the case of database presented in this study, the poor statistical distribution may be attributed to the low quality of data of testing results of specific gravity. Only 28 out of 427 recorded tests had benefited from direct specific gravity measurement (less than 7%). Due to this reason, the statistical indices did not improve even after data cleanup process, which suggests that there is a lack of reliable data for this application.

As for the preconsolidation pressure, high statistical parameters were also observed for the original data. It is due to the significantly high preconsolidation pressure for the layers at shallow depths in association with desiccation rather than the overconsolidation of virgin clay induced by the removal of surcharge. The skewness and kurtosis of the recompression index, *C*_*r*_ is significantly higher compared to those of compression index*, C*_*c*_. The cause of this phenomenon lies in the susceptibility of the undisturbed sample to undergo various disturbances and changes in the stress state. These disturbances occur from the moment sample is extruded from the thin-walled sampler, through its trimming into the consolidation ring, and finally, upon experiencing the seating pressure of the oedometer apparatus. The level of disturbance highly depends on the operator’s skills and competence. These sample disturbances can have adverse effects on the clay structure at the early stages of loading, which leads to a wide range of recompression index values in the overconsolidated range. Once the test pressure exceeds the preconsolidation pressure, there is a higher chance of diminishing the adverse effects on the sample. These observations indicate that the use of unreasonably high preconsolidation pressure and low recompression index based on the existing database without consolidation tests can lead to unexpected excessive settlement in practice.

### Spatial Distribution of Various Parameters

As can be seen in Fig. 8, physical and mechanical soil parameters show differences from east to west of downtown Ottawa. Hence, an arbitrary border was selected to divide the Ottawa area into west and east to further investigate the soil properties between the west and the east of Ottawa. The division point was selected crossing through the middle of downtown Ottawa, the database was separated to the east and west clusters and the average values are presented in Table 5.

**Table 5:** Comparison between the average values of selected parameters from west to east of Ottawa

Parameter	Unit	Average value (west)	Average value (east)	Difference (%)
Preconsolidation pressure ($\sigma_{P}^{\prime}$)	kPa	207	227	8.8
Overconsolidation ratio ($OCR$)	n/a	3.06	3.60	15.0
Initial void ratio (${e}_{o}$)	n/a	1.35	1.63	17.2
Compression index (${C}_{c}$)	n/a	0.897	1.105	18.8
Recompression index (${C}_{r}$)	n/a	0.045	0.045	0.0
Plasticity index ($PI$)	%	22	30	26.7
Undrained shear strength (${S}_{u}$)	kPa	52	53	1.9
Clay percentage ($C$)	%	42	54	22.2

**Fig. 9:**
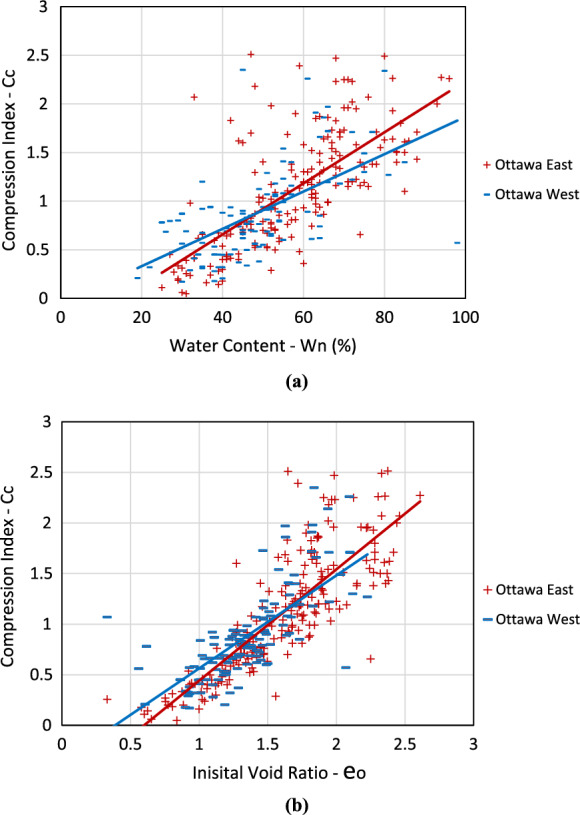
Comparison of the compression index relative to **a** natural water content and **b** initial void ratio, between west and east of Ottawa Valley

**Fig. 10:**
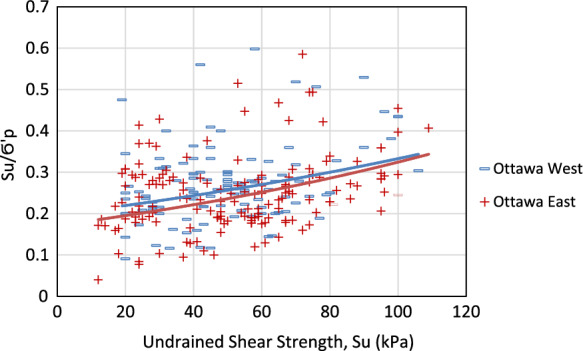
Comparison of the ratio of undrained shear strength to preconsolidation pressure between west and east of Ottawa Valley

As shown in Table 5, the average values of most parameters demonstrate a noticeable difference from east to west of the study area, except for *C*_*r*_ and *S*_*u*_. This is also evident in Fig. 8e–g, as there is no evident distinction between eastern and western areas by looking at the color-coded values for *C*_*r*_ and *S*_*u*_, respectively. One commonly used method by the academia and the industry is to estimate the preconsolidation pressure based on the undrained shear strength of undisturbed clay and plasticity index. Figure 9 displays comparisons of natural water content versus compression index and initial void ratio versus compression index between the east and west regions of Ottawa. The effect of only shear strength was isolated and plotted for the data from both the east and west regions of the chosen border line, as shown in Fig. 10. The region-specific variance in the correlation trend is evident in both figures.

**Fig. 11:**
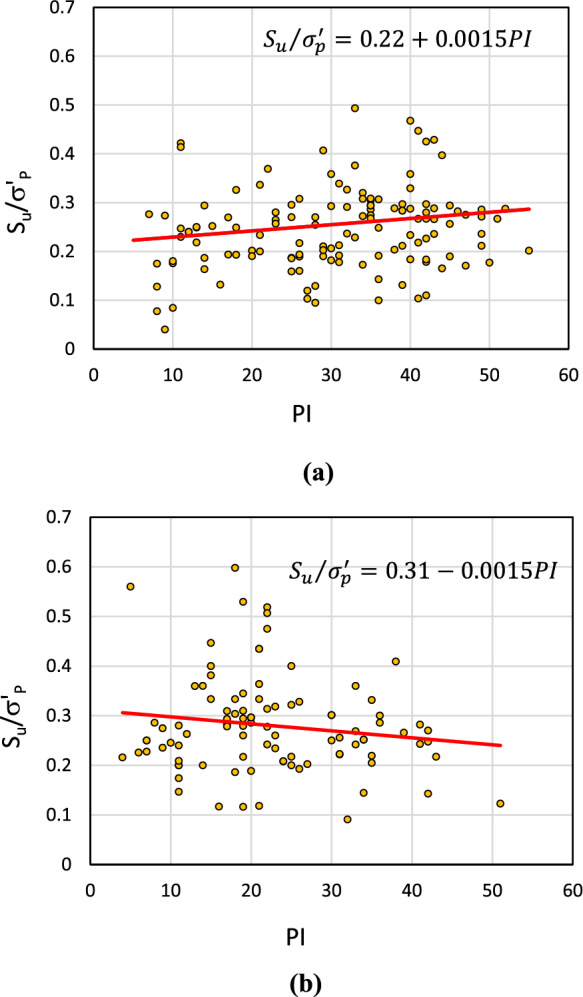
Correlations for estimation of preconsolidation pressure based on undrained shear strength and plasticity index **a** correlation for Ottawa East, **b** correlation for Ottawa West

**Fig. 12:**
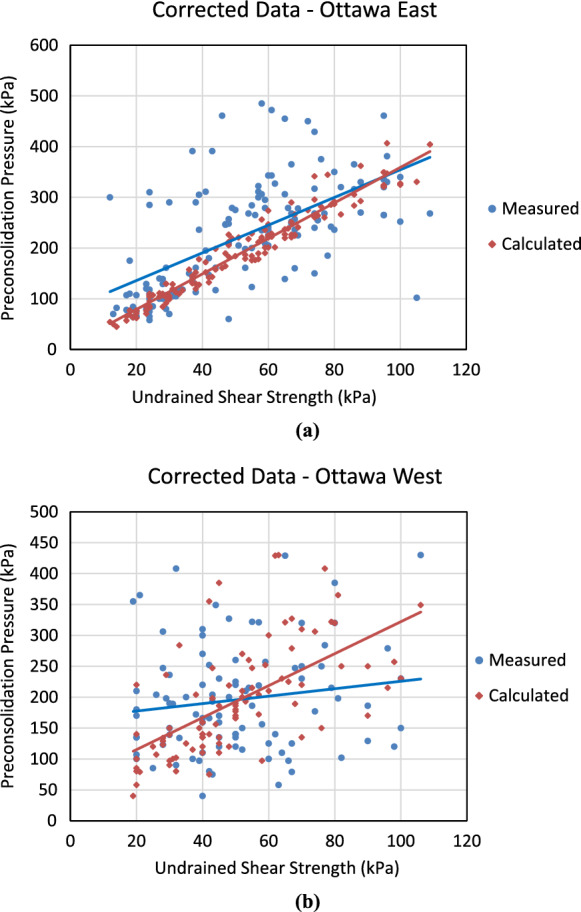
Correlations for the estimation of preconsolidation pressure based on undrained shear strength and plasticity index using the correlation proposed by Leroueil et al. (1983) for **a** Ottawa East and **b** Ottawa West

As shown in Fig. 10, although the *S*_*u*_/${\sigma }_{P}^{\prime}$ ratios show scattered distribution, *S*_*u*_/${\sigma }_{P}^{\prime}$ nonlinearly increases with increasing *S*_*u*_ and no noticeable differences are observed between the western and eastern Ottawa. This is because, as shown in Table 5, the differences in the average ${\sigma }_{P}^{\prime}$ and *S*_*u*_ values for the western and eastern Ottawa region are not significant. On the other hand, noticeably different correlations were observed between east and west of Ottawa Valley when the *S*_*u*_/${\sigma }_{P}^{\prime}$ ratios are plotted against *PI*, as shown in Fig. 11(a) and (b), respectively. These plots highlight the need for region-specific correlations for prediction of preconsolidation pressure of Champlain Sea Clay. It also emphasizes that different approaches are necessary to develop correlations to estimate ${\sigma }_{P}^{\prime}$ using physical and mechanical soil properties of CSC. One of the equations widely used by the industry is offered by Leroueil et al. (1983) which is a correlation between the undrained shear strength, plasticity index, and preconsolidation pressure as shown in Eq. (3). This equation was tested for Ottawa East and Ottawa West separately and is shown in Fig. 12a, b.

3 $${S}_{u}/{\sigma }_{P}^{\prime}=0.2+0.0024PI$$

As illustrated in Fig. 12, the equation proposed by Leroueil et al. (1983) demonstrates a strong correlation for Ottawa East but yields comparatively unsatisfactory results for Ottawa West. Such a behavior in the trend of results demonstrates the necessity of considering spatial distribution of soil parameters when developing any correlations depending on the soil properties used. In other words, it is necessary to adjust the existing correlations through fine-tuning or establish region-specific correlations guided by defined micro-zoning.

For some quick clays, the generated pore-pressure due to shearing is measured twice as high as the peak deviator stress (Mitchell and Soga 2005). Therefore, the ratio of undrained shear strength to effective stress decreases with increasing sensitivity. The magnitude of excess pore-pressure developed during undrained loading depends on the soil fabric, density, existing effective stress, and overconsolidation ratio.

The measured undrained shear strength from triaxial tests may yield different results based on the rate of loading. The undrained shear strength of sensitive clay from laboratory tests is typically lower compared to in-situ (i.e., field) tests because of samples disturbance (Elsawy et al. 2022). The field test methods also affect the shear strength of sensitive clays (Tran et al. 2022). Therefore, in the present study, the undrained shear strength values for both intact and remoulded conditions were obtained solely from field vane tests.

## Limitations of the Research

The entire consolidation testing process, from sampling to deriving the results, is vulnerable to operator bias, which can lead to inherent inconsistencies in the data. Various factors, such as sampling tools, preservation methods, environmental exposure, transportation method, and duration and conditions of storage, can all impact the results. Furthermore, operators' biases performing laboratory tests can influence the results at every stage, from sample extrusion to trimming. It is well-established that sample disturbances can significantly affect preconsolidation pressures (Mataić et al. 2016). Despite acknowledging these influential factors, developing an unbiased method to mitigate the adverse effects of improper sampling, handling, and testing across the entire database was challenging, given that the data was collected over an extended period by numerous site technicians and laboratory operators. Nonetheless, the mathematical analysis employed in this study is reasonably capable of identifying obvious deficiencies, apparent errors, or inconsistencies with the general site conditions.

## Summary and Conclusions

Champlain Sea clay (CSC) poses significant challenges for geotechnical engineers in their practice applications, including excessive consolidation settlements. To address this issue, in the present study, a comprehensive database was created by collecting the existing, accessible, and useable data on the properties of CSC of eastern Canada, focusing on Ottawa region. The data is mostly collected from the reports generated by the industry along with limited information from the published literature. A total number of 509 foundation engineering reports (including MTO reports) have been collected for the Ottawa region and the physical and mechanical property data has been analyzed statistically using skewness and kurtosis. The conclusions obtained from the data analyses are summarized below.

The comprehensive literature indicated that local groundwater typically lowers in associated with rapid urbanization, which is one of the most important contributing factors to the excessive settlement of residential buildings. According to the statistical data analyses, compared to other soil properties, the data skewness and kurtosis were significantly higher for preconsolidation and recompression index. This indicates that the estimation of consolidation settlement with unreasonably high preconsolidation pressure and low recompression index can lead to unexpected excessive consolidation settlement due to the groundwater decline. On the other hand, preconsolidation pressure of Champlain Sea clay can be as low as 24 kPa, therefore errors in the stress history information of the clay can trigger large settlements even for small increase of effective stresses associated with groundwater decline.

The same data was reorganized for the west and east Ottawa Valley with the average values. Noticeably different correlations were observed between east and west of Ottawa Valley, which clearly demonstrates the need for considering spatial distribution of soil parameters when developing any correlations based on the soil properties. This was also supported by the *S*_*u*_/${\sigma }_{P}^{\prime}$ versus *PI* correlations for the data obtained from west and east Ottawa Valley. In addition, better comparison was observed between the measured preconsolidation pressure and those calculated using the correlation proposed by Leroueil et al. (1983) for the data from east Ottawa Valley, when compared to west Ottawa Valley.

The collected data has been cleaned up statistically based on the skewness and kurtosis, which significantly improved the statistical indices, especially ${\sigma }_{P}^{\prime}$, *OCR*, *C*_*c*_ and *C*_*r*_. It is expected that the cleaned-up data can be effectively used for developing reliable correlations to estimate consolidation properties for site-specific data for low-budget projects.

## Data Availability

The datasets generated during and/or analyzed during the current study are publicly available.
